# Testing and Development of Slightly Thick Infant Formula Recipes for Dysphagia Management: An Australian Perspective

**DOI:** 10.1007/s00455-022-10550-1

**Published:** 2023-01-13

**Authors:** Jeanne Marshall, Jordyn Buttsworth, Hannah D. S. Grandt, Madeline Raatz, Annabelle Signorini, Shenali Fernando, Sally Clarke

**Affiliations:** 1 Speech Pathology Department, Queensland Children’s Hospital, Children’s Health Queensland Hospital and Health Service, Brisbane, Australia; 2 School of Health and Rehabilitation Sciences, The University of Queensland, Brisbane, Australia

**Keywords:** Infant feeding, Bottle feeding, Thickened fluids, Dysphagia, Deglutition, Deglutition disorders

## Abstract

Thickened feeds may be useful in supporting improved suck–swallow–breath coordination and airway protection in infants with dysphagia. Unfortunately, the stability of thickened feeds for infant formulas is unpredictable, which makes use of this strategy challenging. This study aimed to propose a set of Level 1 (slightly thick) recipes for Australian infant formulas/thickeners. A secondary aim was to test whether formula could be batch prepared. A set of powdered, ready-to-feed, and specialized formulas were mixed with two thickening products (Aptamil Feed Thickener® and Supercol®) and tested at 5-, 10-, 15-, 20-, 25-, 30-, and 45-min intervals using the International Dysphagia Diet Standardization Initiative (IDDSI) Flow Test. Formula/thickener samples were mixed following manufacturer instructions, but recipes were adapted to determine an ideal recipe for Level 1 (slightly thick) consistency that would be maintained over a feed. Samples were refrigerated, reheated after 12 h, and retested. Each combination was tested six times. Overall, 1,353 IDDSI Flow Tests were conducted using 14 formula/thickener combinations. In all combinations, recipe alterations were made using metric spoon measurements as opposed to the manufacturer-provided scoop. All samples were most variable at the 5-min timepoint. Formulas thickened with Supercol® generally reached a more stable consistency by 10 min, whereas formulas thickened with Aptamil Feed Thickener® were more stable by 15 min. Samples tested after 12 h were more variable with Aptamil Feed Thickener®. This study provides practical recommendations for clinicians working with infants requiring thickened feeds for dysphagia management. Further study under controlled laboratory conditions is required.

## Introduction

Many fragile and medically complex neonates and infants may have difficulties with suck–swallow–breath coordination, which challenges their ability to feed safely and efficiently. Infants with oropharyngeal dysphagia secondary to suck–swallow–breath incoordination may experience penetration, aspiration, and/or desaturation, which can induce further medical complications, placing them at risk of harm [1–3]. These infants may also be at risk of poor growth and feeding tube placement [4, 5], as well as prolonged hospital admissions [6]. In the longer term, early feeding difficulties may predispose them to experiencing chronic feeding problems [1, 5, 7].

In adult patients, increasing fluid thickness is a relatively common means by which speech pathologists support patients with penetration/aspiration. Use of a slower flowing liquid can provide the patient more time to coordinate their swallow and improve bolus cohesion [8]. Although not as commonly applied, altering the thickness of infant formula has also been demonstrated to reduce nipple flow rate [9]. In theory, this allows more time for swallow coordination, reducing physiological compromise and aspiration risk during feeding and permitting ongoing oral intake [10, 11]. For infants that require thickened fluids, IDDSI Level 1 (slightly thick) is often recommended, as this level of thickened fluid is more able to flow through a teat [12] as compared to thicker fluids (e.g., Level 2, mildly thick), although thicker fluids may be recommended where indicated. Parents generally have a perception of improved feeding outcomes and quality of life when thickener is used [13, 14], although thickener is reported to be challenging to manage in a home environment [14].

Managing infant dysphagia using thickener is challenging for clinicians and families, complicated by a range of different issues. Unfortunately, there are multiple factors that influence fluid thickness, including the type of base fluid used (i.e., breastmilk, powdered formula, or ready-to-feed formula), the type of thickener used (i.e., starch vs. gum based), the time elapsed since mixing, the temperature of the fluid, and the way in which the formula/thickener combination was mixed [11, 15–18]. Several studies have also concluded that the rate and consistency of thickening varies between infant formulas when combined with different thickeners [10, 19, 20]. Faster flowing bottle nipples are often required to accommodate the thicker fluid [11, 21], which are not always readily available to parents. In addition to factors affecting fluid thickness and the availability of appropriate equipment, there are limited safe thickener products available that are for use in infants less than 1 year of age, who are predominantly breast or formula fed. Ng and colleagues (2022) recently explored the influence of time on thickened infant formulas, testing these at baseline (2 min after mixing), 1- and 24-h timepoints for Australian formula products (combined with Aptamil® Feed Thickener and Supercol®). This research found that some thickened formula samples mixed according to recommendations were thinner than Level 1 (slightly thick) at baseline, whereas some samples were thicker than Level 1 (slightly thick) after 24 h. They also found that cold samples were significantly thicker than room-temperature samples. Although this study did collect useful information regarding Australian products, it did not fully explore the changes in thickness between 0 and 1 hour, which is the period during which an infant would typically take the feed. These challenges prompted the current study into thickened infant formula preparation for an immediate feed.

The International Dysphagia Diet Standardization Initiative (IDDSI) was founded in 2013, with an aim to propose global standards for terminology and definitions regarding foods and fluids [22, 23]. As part of this process, the IDDSI Flow Test was developed as a clinical bedside assessment to test the thickness of fluids [23]. This test involves filling a 10-ml syringe with 10 ml of the testing liquid and measuring the amount of liquid that flows through the syringe in a period of 10 s. A Level 1 (slightly thick) fluid results in between 1 and 4 ml of fluid remaining in the syringe after 10 s has elapsed [23]. This assessment is commonly used in clinical practice and has been used in recent studies regarding thickened fluids in infants [10, 17, 24].

The primary aim of this study was to use the IDDSI Flow Test to propose a set of ideal recipes for thickened infant formulas at Level 1 (slightly thick) consistency using Australian products, including describing ideal formula to thickener ratios, mixing instructions, and a timeframe for feeding when the thickened formula met the requirements for Level 1 (slightly thick). A secondary aim was to assess whether formula/thickener combinations could be stored and reheated for later feeding without excessive changes to thickness. It was hypothesized that there would be variability across different formula/thickener combinations and that these would continue to thicken over time (i.e., that batch preparation would not be feasible).

## Methods

### Infant Formulas and Thickeners

The infant formula products used for this study were selected based on frequency of use in Australian settings, as per the experience of the research team. The research team consisted of speech pathologists working in a tertiary hospital setting, with between 2 and 20 years of clinical experience. Two undergraduate students (Authors 2 and 3) also contributed to this study. The speech pathology team (Authors 1, 4, 5, and 6) agreed upon which products to include for testing in this study based on their experience of commonly used products. “Infant” formulas refer to those advertised as suitable for children aged 0–12 months. Formulas tested are listed in Table 1. Powder formula samples were prepared according to manufacturer instructions. Formula powder was added to water, shaken for 30 s [17, 25], and allowed to sit for at least 2 min to allow for settling of any aeration [10]. Ready-to-feed formulas were gently shaken for 10 s before opening as per the product instructions.

**Table 1:** Products used in testing

Type	Registration owner	Brand name
Standard formulas	Nutricia	Aptamil® Gold + 1
	Alula	S-26® Original Newborn
Ready-to-feed formulas	Alula	S-26® Gold Newborn Ready to Feed
	Nutricia	Aptamil® Gold + Ready to Feed
	Nutricia	Infatrini® Ready to Feed
Specialized formulas	Nutricia	Aptamil® Gold + Pepti-Junior®
	Nutricia	Neocate® Gold
Thickeners	Nutricia	Aptamil® Feed Thickener
	Supercol Australia	Supercol® for Swallowing Difficulties (Dysphagia)

The thickeners used were powdered thickeners: Aptamil® Feed Thickener (Nutricia) and Supercol® for Swallowing Difficulties (Dysphagia) (Supercol Australia) (referred to as “Supercol®” hereafter for readability). These are the only products in Australia available for use in infants under one year of age. Aptamil® Feed Thickener is made of carob bean gum, and may contain allergens such as milk or soy [26]. Supercol® is a guar gum-based thickener that is allergy free [27]. Both are described as being safe for use in infants from birth but are not suitable for use in premature infants. All products were within use by dates and used according to expiry date instructions.

### IDDSI Flow Test

The IDDSI Flow Test was used to measure the thickness of each thickened infant formula sample over time [23]. In accordance with IDDSI recommendations, 10-ml syringes (slip tip) with a 61.5-mm syringe length (from 0 to 10-mL line) were used to conduct the flow tests. Two syringes were used for each test. The experimenter extracted slightly more than 10 ml from the middle of each mixed sample to allow for excess aeration and any settling. Exactly 10 ml of thickened infant formula was then transferred to the test syringe, which was stopped using the experimenter’s finger. The experimenter released their finger and a 10-s timer was started simultaneously as per the IDDSI Flow Test instructions. At the end of 10 s, the experimenter’s finger was replaced and the amount of remaining thickened infant formula in the test syringe to the nearest 0.2 ml was recorded. Data were recorded manually in Microsoft Excel for later analysis.

### Procedure Development

**Fig. 1:**
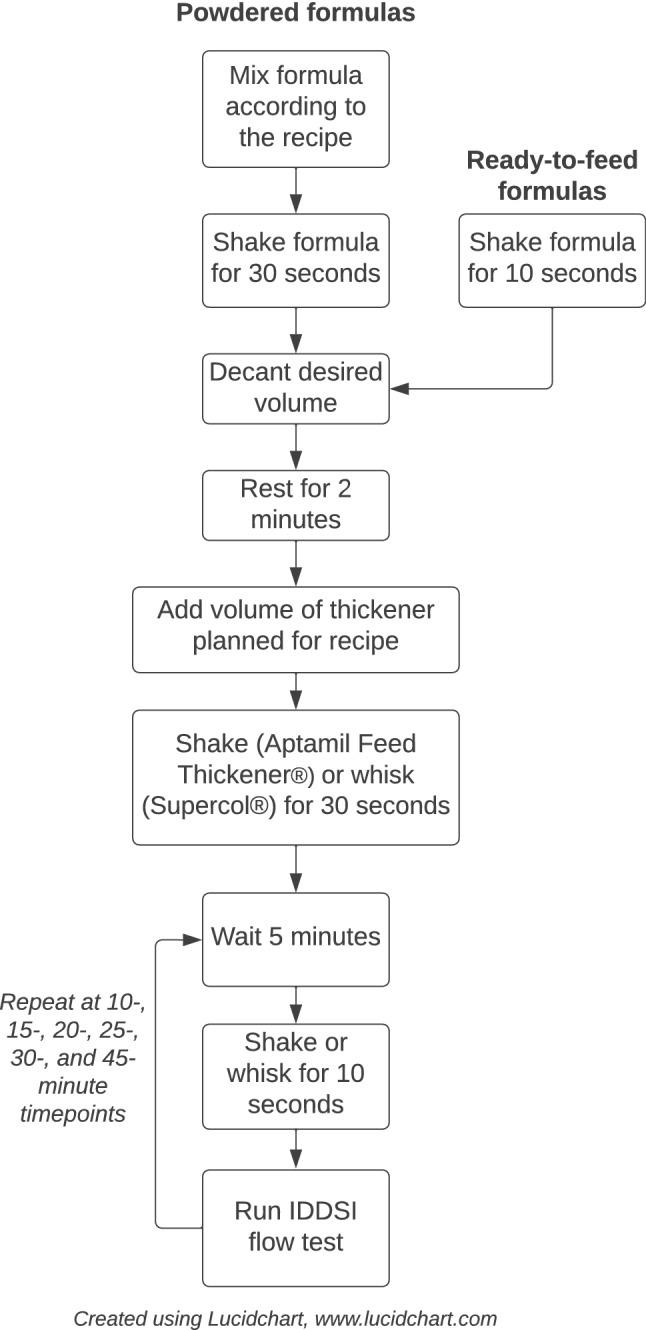
Procedure flowchart

All samples were prepared by two research assistants and/or the primary author over several months. The three testers worked together to refine the methodology and ensure calibration of testing. The final methods for thickened formula preparation are illustrated in (Fig. 1).

For each formula/thickener combination, initially, each sample was prepared according to the instructions using the scoop or recommendations provided by the manufacturer. Mixing methods used (i.e., by hand shaking or using a whisk) were employed to closely mimic the procedure used by parents in the home environment, rather than utilizing equipment such as a blender. The temperature of each sample was measured prior to commencement of testing to ensure that it was at room temperature (23.7 °C–25.3 °C). Room temperature was used for testing as this was the most commonly presented temperature for infant formulas in our work setting. Six repetitions were conducted for each formula/thickener combination at 5-, 10-, 15-, 20-, 25-, 30-, and 45-min timepoints to examine the consistency of thickening and to determine an appropriate timeframe during which the fluid remained at Level 1 (slightly thick). Six repetitions were employed in line with previous research in Australia [10]. The timeframe of 45 min was selected as this was considered a potential timeframe during which an infant might consume a feed, allowing for interruptions (e.g., pauses for burping). In many cases, using the manufacturer-provided scoop produced a result that became too thick in too short a period or remained a thin fluid, neither of which would be functional for use in feeding an infant requiring Level 1 (slightly thick) feeds. Where a slightly thick result was unable to be achieved across more than half (4/7) of the timepoints in each trial (i.e., the formula/thickener combination was either consistently too thin or too thick), the trial was discontinued, and recipe adjustments were made according to a pre-conceived process. As these adaptations were product dependent, they are described in the thickener-specific sections below. To support these adaptations and for reproducibility of recipes, metric spoon measures of each powdered thickener were weighed five times using a digital scale to two decimal places, with a mean and standard deviation presented in Table 2.

**Table 2:** Weights of metric spoon measures for powdered thickener

Measure	Aptamil®*	Supercol®
Manufacturer’s scoop	4.10 g (± 0.09)	0.42 g (± 0.01)
1 Tbsp (20 ml)	10.81 g (± 0.08)	13.88 g (± 0.33)
1/2 Tbsp (10 ml)	5.12 g (± 0.03)	6.33 g (± 0.12)
1 tsp (5 ml)	2.34 g (± 0.02)	2.95 g (± 0.07)
1/2 tsp (2.5 ml)	1.00 g (± 0.02)	1.24 g (± 0.02)
1/4 tsp (1.25 ml)	0.57 g (± 0.02)	0.68 g (± 0.01)
1/8 tsp (0.63 ml)	0.35 g (± 0.01)	0.44 g (± 0.01)

^*^Aptamil® Feed Thickener; Tbsp = tablespoon; Tsp = teaspoon

An additional test was conducted 12 h after the preparation of the initial sample for each final recipe derived to determine whether formula/thickener combinations could be mixed in batches. Formula/thickener combinations were refrigerated for the 12-h period. At 12 h (± 30 min), the formula/thickener combinations were shaken for 10 s and reheated to room temperature (23.7–25.3 °C). Formula was reheated by placing the sample in warm water and agitating the liquid gently every 30 s until room temperature was achieved. Temperature measurements were captured using a digital kitchen thermometer placed in the middle of the sample. An IDDSI test was conducted across all six samples at this timepoint for each formula/thickener combination.

#### Aptamil® Feed Thickener

All formula samples for Aptamil® Feed Thickener trials were prepared in 100-ml volumes**.** Following preparation and settling time, Aptamil® Feed Thickener was added to the formula according to manufacturer’s instructions to obtain a Level 1 (slightly thick) sample. As per Fig. 1, all formula/thickener samples were shaken for a further 30 s to integrate the thickener. At each timepoint, samples were shaken for 10 s prior to conducting the IDDSI Flow Test to maximize homogeneity of thickness [10].

All trials commenced according to instructions with use of the manufacturer’s scoop (mean weight = 4.10 g). For failed trials, where formula/thickener combinations became too thick (i.e., above Level 1, e.g., mildly thick), the amount of thickener was decreased to 1 metric teaspoon (mean weight = 2.34 g) and increased/decreased by half a teaspoon from that point until a desired recipe was reached. If the thickened formula was too thin (i.e., Level 0, a thin fluid) then the amount of thickener was increased to a half metric tablespoon (mean weight = 5.12 g) and increased/decreased by half a teaspoon from that point until a desired recipe was reached. The batch number of each product was systematically recorded.

#### Supercol® for Swallowing Difficulties (Dysphagia)

Formula/thickener combinations using Supercol® were initially prepared using a standard metric 1/4 teaspoon (1.25 ml, Supercol® mean weight = 0.68 g) measurement. According to Supercol® Australia [27], 1/4 teaspoon of Supercol® thickener is equivalent to 2 scoops of Supercol® thickener using the provided scoop. This amount of thickener is recommended for formula volumes of 200 ml. Where possible, the study aimed to devise recipes using the smallest possible fluid volumes to the nearest 50 ml, without resorting to impractically small amounts of Supercol®. Final recipes are therefore representative of the smallest possible volume trialed (to the nearest 50 ml).

To facilitate mixing of formula/thickener samples, Supercol® was added gradually to the formula and whisked simultaneously as per the manufacturer instructions, using a hand whisk. The initial sample was whisked for 30 s. Samples were whisked again for 10 s prior to conducting each IDDSI Flow Test at each timepoint to maximize homogeneity of thickness. Where formula/thickener samples were too thick, the amount of Supercol® thickener added was reduced by 1/8 teaspoon (mean weight = 0.44 g) or the volume of the sample was systematically reduced by 50 ml until a consistent thickening pattern to Level 1 (slightly thick) was observed. Where samples were too thin, the amount of Supercol® thickener added was increased by 1/8 teaspoon or the volume of formula used was decreased by 50 ml.

At times, formulas mixed with Supercol® blocked the syringe, preventing fluid flow. Where this occurred, a new sample was immediately withdrawn with a clean syringe and the flow test was repeated.

### Data Analysis

All data were recorded using Microsoft Excel. Statistical analyses were conducted using SPSS (Version 27). Only the data from the final recipes derived were used in analysis and are included in this manuscript. The mean, standard deviation (SD), and coefficient of variation (CV) were calculated for each timepoint using the data collected across the six trials. The CV was arbitrarily categorized into four levels in a similar manner to other health-based research [28, 29]: low variability (CV < 0.1), moderate variability (CV 0.1 to < 0.2), high variability (CV 0.2 to < 0.3), and very high variability (CV > 0.3). For every formula/thickener combination, the mean values for each timepoint were plotted on a graph using a logarithmic scale, from which a logarithmic equation was deduced. This logarithmic equation was used to calculate the time during which the fluid remained at Level 1 thickness (i.e., with the equation plotted for y = 1 and y = 4), as presented in Table 3 and Table 4. A one-way between groups analysis of variance (ANOVA) with post hoc comparisons using a Tukey HSD test was conducted to explore patterns of thickening over time and to determine whether any one formula/thickener combination thickened at a slower rate than any others. Post hoc testing results from the ANOVA were examined using a conservative p-value of < 0.01.

**Table 3:** Mean IDDSI Level of thickened infant formulas with Aptamil® Feed Thickener

Formula name	Formula volume	Thickener volume	Mean IDDSI Level* (± SD)							Predicted time at Level 1 (in mins)^
			5 min	10 min	15 min	20 min	25 min	30 min	45 min	
Standard formulas										
Aptamil® Gold + 1	100 ml	1½ tsp	0.63 (± 0.23) *CV* = *0.37*	1.63 (± 0.29) *CV* = *0.18*	2.27 (± 0.16) *CV* = *0.07*	2.80 (± 0.31) *CV* = *0.11*	3.10 (± 0.21) *CV* = *0.07*	3.40 (± 0.22) *CV* = *0.06*	3.73 (± 0.11) *CV* = *0.09*	6.3 to 48.2
S-26® Original Newborn	100 ml	1½ tsp	0.40 (± 0.28) *CV* = *0.70*	1.43 (± 0.46) *CV* = *0.32*	1.93 (± 0.21) *CV* = *0.11*	2.43 (± 0.29) *CV* = *0.12*	2.67 (± 0.33) *CV* = *0.12*	3.03 (± 0.45) *CV* = *0.15*	3.37 (± 0.34) *CV* = *0.10*	7.5 to 65.2
Ready-to-feed formulas										
Aptamil® Gold + RTF	100 ml	1½ tsp	0.17 (± 0.15) *CV* = *0.90*	0.87 (± 0.24) *CV* = *0.28*	1.6 (± 0.44) *CV* = *0.27*	2.23 (± 0.32) *CV* = *0.14*	2.27 (± 0.37) *CV* = *0.16*	2.4 (± 0.46) *CV* = *0.19*	2.87 (± 0.50) *CV* = *0.17*	9.6 to 99.3
S-26® Gold Newborn RTF	100 ml	1½ tsp	0.10 (± 0.11) *CV* = *1.10*	0.93 (± 0.10) *CV* = *0.11*	1.50 (± 0.33) *CV* = *0.22*	1.87 (± 0.16) *CV* = *0.09*	2.13 (± 0.21) *CV* = *0.10*	2.50 (± 0.28) *CV* = *0.11*	2.67 (± 0.24) *CV* = *0.09*	10.2 to 115.9
Nutricia Infatrini® RTF	100 ml	1½ tsp	0.50 (± 0.11) *CV* = *0.22*	1.33 (± 0.24) *CV* = *0.18*	2.10 (± 0.17) *CV* = *0.08*	2.57 (± 0.37) *CV* = *0.14*	3.07 (± 0.16) *CV* = *0.05*	3.20 (± 0.40) *CV* = *0.13*	3.73 (± 0.35) *CV* = *0.09*	7.2 to 51.0
Specialized formulas										
Aptamil® Gold + Pepti-Junior®	100 ml	1½ tsp	0.13 (± 0.21) *CV* = *1.55*	0.90 (± 0.33) *CV* = *0.37*	1.27 (± 0.33) *CV* = *0.26*	1.87 (± 0.35) *CV* = *0.19*	2.00 (± 0.59) *CV* = *0.13*	2.13 (± 0.30) *CV* = *0.14*	2.20 (± 0.33) *CV* = *0.15*	10.8 to 200.9
Neocate® Gold	100 ml	1½ tsp	0.20 (± 0.18) *CV* = *0.89*	1.2 (± 0.18) *CV* = *0.15*	1.60 (± 0.40) *CV* = *0.25*	2.17 (± 0.15) *CV* = *0.07*	2.26 (± 0.24) *CV* = *0.11*	2.33 (± 0.33) *CV* = *0.14*	2.80 (± 0.33) *CV* = *0.12*	8.9 to 113.4

^*^A result between 1 and 4 ml was considered to be a Level 1 (slightly thick) thickness, consistent with IDDSI guidelines

^A logarithmic equation was used to calculate the time during which the fluid remained at Level-1 thickness (i.e., with the equation plotted for y = 1 and y = 4)

Abbreviations: IDDSI = International Dysphagia Diet Standardization Initiative, SD = Standard Deviation, CV = Coefficient of variation, tsp = standard metric teaspoon, tbsp = standard metric tablespoon, mins = minutes, RTF = ready to feed

Coefficient of variation was calculated using SD/mean, applying the ratings < 0.1 (low), 0.1 to < 0.2 (medium), 0.2 to < 0.3 (high), > 0.3 (very high)

**Table 4:** Mean IDDSI Level of thickened infant formula with Supercol®

Formula name	Formula volume	Thickener amount	Mean IDDSI Level* (± SD)							Predicted time at Level 1 (in mins)^
			5 min	10 min	15 min	20 min	25 min	30 min	45 min	
Standard formulas										
Aptamil® Gold + 1	150 ml	1/8 tsp	0.20 (± 0.23) *CV* = *1.3*	1.07 (± 0.10) *CV* = *0.10*	1.63 (± 0.29) *CV* = *0.18*	1.83 (± 0.23) *CV* = *0.13*	2.00 (± 0.13) *CV* = *0.06*	2.20 (± 0.25) *CV* = *0.11*	2.37 (± 0.27) *CV* = *0.11*	9.5 to 185.6
S-26® Original Newborn	150 ml	1/8 tsp	0.67 (± 0.21) *CV* = *0.31*	1.27 (± 0.39) *CV* = *0.31*	1.87 (± 0.52) *CV* = *0.28*	2.40 (± 0.36) *CV* = *0.15*	2.23 (± 0.27) *CV* = *0.12*	2.33 (± 0.39) *CV* = *0.17*	2.77 (± 0.46) *CV* = *0.17*	6.7 to 147.0
Ready-to-feed formulas										
Aptamil® Gold RTF	200 ml	1/4 tsp	0.97 (± 0.32) *CV* = *0.33*	2.10 (± 0.28) *CV* = *0.13*	2.47 (± 0.37) *CV* = *0.15*	2.93 (± 0.16) *CV* = *0.06*	3.07 (± 0.10) *CV* = *0.03*	3.23 (± 0.41) *CV* = *0.13*	3.23 (± 0.29) *CV* = *0.09*	4.1 to 65.6
S-26® Gold Newborn RTF	150 ml	1/8 tsp	0.20 (± 0.22) *CV* = *1.10*	0.90 (± 0.24) *CV* = *0.27*	1.3 (± 0.33) *CV* = *0.25*	1.53 (0.47) *CV* = *0.30*	1.67 (± 0.24) *CV* = *0.15*	1.80 (± 0.38) *CV* = *0.21*	2.13 (± 0.47) *CV* = *0.22*	11.6 to 360.9
Nutricia Infatrini® RTF	150 ml	1/8 tsp	0.47 (± 0.35) *CV* = *0.75*	1.53 (± 0.41) *CV* = *0.27*	2.03 (± 0.39) *CV* = *0.19*	2.77 (0.48) *CV* = *0.17*	2.83 (± 0.61) *CV* = *0.22*	3.27 (± 0.89) *CV* = *0.27*	3.82 (± 1.22) *CV* = *0.32*	7.1 to 50.1
Specialized formulas										
Aptamil® Gold + Pepti-Junior®	300 ml	3/8 tsp	1.03 (± 0.23) *CV* = *0.65*	1.9 (± 0.21) *CV* = *0.11*	2.27 (± 0.48) *CV* = *0.21*	2.57 (± 0.43) *CV* = *0.17*	2.7 (± 0.59) *CV* = *0.22*	2.77 (± 0.52) *CV* = *0.19*	2.63 (± 0.29) *CV* = *0.11*	3.5 to 156.4
Neocate® Gold	200 ml	1/4 tsp	2.10 (± 0.37) *CV* = *0.18*	3.20 (± 0.47) *CV* = *0.14*	3.30 (± 0.39) *CV* = *0.12*	3.30 (± 0.41) *CV* = *0.13*	3.73 (± 0.45) *CV* = *0.12*	3.47 (± 0.33) *CV* = *0.09*	4.07 (± 0.60) *CV* = *0.15*	0.9 to 42.4

^*^A result between 1 and 4 ml was considered to be a Level 1 (slightly thick) thickness, consistent with IDDSI guidelines

^A logarithmic equation was used to calculate the time during which the fluid remained at Level 1 thickness (i.e., with the equation plotted for y = *1 and y* = 4)

Abbreviations: IDDSI = International Dysphagia Diet Standardization Initiative, SD = Standard Deviation, CV = Coefficient of variation, tsp = standard metric teaspoon, tbsp = standard metric tablespoon, mins = minutes, RTF = ready to feed

Coefficient of variation was calculated using SD/mean, applying the ratings < 0.1 (low), 0.1 to < 0.2 (medium), 0.2 to < 0.3 (high), > 0.3 (very high)

## Results

Ideal recipes were developed over several testing iterations and are presented using metric spoon measures in Tables 3 and 4. A total of 1,353 IDDSI Flow Tests were conducted using 14 infant formula/thickener combinations, with means, SDs, and CVs presented. Data regarding testing at the 12-h timepoint are presented in Table 5.

**Table 5:** Formula thickness after 12 h using final recipes (warmed to room temperature)

Formula name	Aptamil® thickener Mean (± SD) *CV*	Supercol® thickener Mean (± SD) *CV*
Aptamil® Gold + 1	3.8 (± 0.22) *CV* = *0.06*	2.4 (± 0.28) *CV* = *0.12*
S-26® Original Newborn	4.1 (± 0.30)* *CV* = *0.07*	3.0 (± 0.30) *CV* = *0.07*
Aptamil® Gold RTF	3.33 (± 0.41)* *CV* = *0.12*	2.17 (± 0.78) *CV* = *0.36*
S-26® Gold Newborn RTF	3.47 (± 0.47) *CV* = *0.13*	3.30 (± 0.33) *CV* = *0.10*
Nutricia Infatrini® RTF	5.10 (± 0.45)* *CV* = *0.09*	3.33 (± 0.35) *CV* = *0.11*
Aptamil® Gold + Pepti-Junior®	2.80 (± 0.40) *CV* = *0.14*	2.4 (± 0.25) *CV* = *0.10*
Neocate® Gold	3.10 (± 0.30) *CV* = *0.10*	3.0 (± 0.18)* *CV* = *0.06*

*Result is significantly different to final thickness at 45-min timepoint for this formula/thickener combination (p < 0.01); RTF = ready to feed

In general, formulas thickened with Aptamil® Feed Thickener reached Level 1 (slightly thick) between 6 and 10 min. A standard recipe of 1.5 teaspoons/100-ml formula was found to be the most consistent thickener volume, regardless of the formula used. Examination of the CV, however, indicated medium to very high variability in thickness until the 15-min timepoint. This result was supported by the outcomes of ANOVA testing, where post hoc analysis revealed significant differences between timepoints were most common across the 5-, 10-, and 15-min marks (p < 0.01). Additionally, three formula/thickener combinations were significantly thicker at the 12-h timepoint despite reheating (*p* < 0.01) (see Table 5). Apart from S-26® Original Newborn powdered formula and Infatrini® ready-to-feed, however, most of the formula/thickener combinations remained within the acceptable range for Level 1 (slightly thick) thickness at this 12-h timepoint.

Formulas thickened with Supercol® reached ideal thickness between 1 and 11 min. Recipes were more variable than those derived for Aptamil® feed thickener, with differing formula volumes and amounts of Supercol® used across different products, as is described in Table 4. Although these formula/thickener combinations did continue to thicken, analysis of the ANOVA post hoc testing outcomes suggested they did so at a slower rate than those thickened with Aptamil® Feed Thickener, with significant differences most consistently observed at the 5-min timepoint only (*p* < 0.01). Examination of the CV also indicated very high variability at the 5-min timepoint. Apart from the S-26® Original Newborn powdered formula/Supercol® combination, the CV was more acceptable from the 10-min timepoint. Of interest, compared to the samples mixed with Aptamil® Feed Thickener, samples mixed with Supercol® often became slightly thinner at the 12-h timepoint, although this difference was not statistically significant, with the exception of the Neocate® Gold/Supercol® combination (mean at 45 min = 4.1; mean at 12 h = 3.0; *p* < 0.01). Blockage of the syringe and retesting occurred on 17 occasions using the Supercol® thickener and formula combinations.

Across both thickener and all formula types, formula/thickener combinations were most variable at the 5-min timepoint. Where the standard formulas and specialized formulas remained at low-medium consistency by the end of the testing period at the 45-min timepoint, the ready-to-feed formulas were more variable in their presentation, with CVs ranging from 0.06 to 0.32 (low to very high). With the exception of the S-26® Gold Newborn Ready-To-Feed/Supercol®, which had a high CV, the other formula/thickener combinations had low-medium CVs at the 12-h timepoint, ranging from 0.06 to 0.14.

## Discussion

This study explored the development of consistent recipes for Level 1 (slightly thick) infant formulas, examining ideal formula to thickener ratios and the influence of time. The findings of this study align with prior research, in that different formula/thickener combinations resulted in different patterns of thickening despite being tested under consistent conditions [10, 18, 19]. Testing of reheated Level 1 (slightly thick) formula/thickener combinations at the 12-h timepoint yielded generally acceptable results. This work will provide clinicians with practical guidelines to support use of thickened formula for infants that may require it.

There is evidence that thickeners are helpful in reducing penetration and pulmonary admissions for some children [30]; however, current literature suggests that further experimental studies are required regarding their efficacy [31, 32]. This study provided further evidence of the challenges of using thickened infant formulas in practice, with thickness influenced by time, type of formula used, and type of thickener used. It was unclear as to why formulas with similar ingredients presented differently in terms of thickening patterns, and more in-depth exploration of the interaction between different products is warranted to explain this phenomenon. As per previous literature in this area, due to the complexity of the variables contributing to thickness, thickened fluids should only be recommended as a last resort in the management of infant feeding [17]. Other strategies to support reducing fluid flow rate (e.g., external pacing, side-lying positioning, employing a slower flowing nipple) should ideally be considered prior to the use of thickened infant formula.

Despite the development of recipes that were considered to be acceptable at Level 1 (slightly thick), multiple challenges were observed and should be considered in using this as a management strategy. Statistical analyses indicated significant variability in thickness until the 15-min timepoint for formulas mixed with Aptamil® Feed Thickener and to the 10-min timepoint for those mixed with Supercol®. A previous study identified that it takes at least 10 min for formula thickened with a starch-based thickener to reach a stable thickness, regardless of the amount of thickener used [25]. As a rule of thumb and consistent with previous research, clinicians should therefore consider waiting a minimum of 10 min after mixing any infant formula/thickener combination before commencing feeding. Additionally, this study only tested formula/thickener combinations until the 45-min timepoint, which conservatively suggests that with the allowance of the initial 10 min, the formula would remain at a Level 1 (slightly thick) consistency for approximately 30 min after it reaches appropriate thickness. The logarithmic equations suggest some formula/thickener combinations may maintain appropriate thickness levels for a longer period; however, these are models only, so should be interpreted with caution (see Tables 3 and 4). In general, compared to the powdered formulas, the ready-to-feed formulas demonstrated higher variability in consistency of testing across timepoints in the first 45 min. This is congruent with previous research that has observed variability in ready-to-feed formulas when mixed with powdered thickener [16] and is an additional consideration. Finally, although it was a rare occurrence, blockage of the syringe did occur on some occasions with the Supercol® thickener and should be monitored during feeding with this product. Therefore, although this study was able to generate a set of consistent recipes as guidance for clinical practice, clinicians are advised to consider the general variability of thickened feeds and use thickeners with caution in infants. Clinicians should also consider ongoing calibration of their recipes by periodically retesting hand-thickened formulas, and training families in the use of flow testing for their own quality control.

This experiment required the testers to shake the formula/thickener combinations for 10 s prior to completing flow tests at each timepoint, in a similar method to previous research [10]. This step was included to maximize the homogeneity of thickness for testing by preventing settling of the thickener. This settling effect was observed during initial iterations of procedural design. In an actual feed, however, the feeder does not pause to shake the bottle every 5 min, so there may be a settling effect and variances to thickness in clinical practice. In a natural feeding situation, the feeder is encouraged to gently shake the bottle during pauses to ensure ongoing mixing of the thickener and formula for the duration of the feed.

Mixing fluids in large volumes, as is recommended in the ideal recipes proposed, can be challenging in infants with dysphagia, who may only be taking small volumes due to efficiency issues or who have time limitations on feeds for safety purposes. It should be noted that thickener recipes are not linear—they cannot simply be halved or doubled to mix with different volumes of formula. Previous research has demonstrated that simply doubling the amount of powder and fluid may not result in the same fluid thickness [33, 34]. The same cautions apply to the recipes presented in this paper. The 12-h batch testing of this formula indicated that most of these formula/thickener combinations can be prepared in batches and reheated to room temperature, while maintaining a Level-1 (slightly thick) result. Gosa and Dodrill (2016) [19] demonstrated that formula/thickeners in their experiment reheated at 3-h post-mixing had variable results on a line spread test. Although the present study does provide some useful information, further research in a controlled laboratory environment is required.

### Limitations

While this study provides useful evidence regarding Australian formulas and thickening agents, there is the possibility of human error. This study used the IDDSI Flow Test to test the thickness of various infant formula/thickener combinations rather than a rheometer, which is recommended for examining thickened fluid properties [11]. The 10-s IDDSI Flow Test utilized throughout experimentation relies on accurate timing of the experimenter for stopping the flow, and measurements were taken visually by the experimenter, relying on accurate judgment and eyesight. As the processes employed in this study are subject to human error, the suggested recipes are intended as a guide for clinical practice. It is imperative that clinicians use their own expertise and conduct an IDDSI Flow Test at bedside if there is any doubt of suitability of a formula/thickener combination for the individual infant.

Besides the ratio of formula to thickener and the impact of time, it is known that temperature has an impact on fluid thickness [10, 19]. Throughout the experimental stage of the current study, the temperature of samples was monitored and remained at room temperature across all days of experimentation (23.7–25.3 °C). It is unclear whether this impacted the thickness of the samples as there was no equipment in place to control or measure temperature continuously throughout testing. Additionally, this study used hand shaking and whisking to mix formula and thickener samples, as per manufacturer instructions, and to mimic what might be done in a home environment. Other research has demonstrated that variability in thickness may occur with different mixing methods [17], and clinicians should consider that different results may occur if the proposed recipes are mixed using different methods. These are all variables to consider in future research in a well-resourced laboratory.

## Conclusion

The results from this study provide practical recommendations for health care professionals working with infants who require Level 1 (slightly thick) formula for the management of dysphagia. Results proposed ideal formula/thickener ratios for room-temperature fluids and suggest waiting at least 10 min after initial mixing for thickened infant formula to reach an ideal thickness. Results also suggest that thickened formula can be reheated to room temperature to achieve a previously appropriate Level-1 (slightly thick) result. Given the variability of thickened infant formula, other management strategies to support reducing flow rate (i.e., external pacing, side-lying positioning, use of a slow flow nipple) should be considered prior to using powdered thickener.
